# Loading pattern of postoperative hallux valgus feet with and without transfer metatarsalgia: a case control study

**DOI:** 10.1186/s13018-017-0622-z

**Published:** 2017-07-25

**Authors:** Xiang Geng, Dichao Huang, Xu Wang, Chao Zhang, Jiazhang Huang, Xin Ma, Li Chen, Chen Wang, Junsheng Yang, Heng Wang

**Affiliations:** Department of Orthopedics, Huashan Hospital, Fudan University, 12 Middle Wulumuqi Road, Shanghai, 200040 China

**Keywords:** Gait analysis, Loading pattern, Pedography, Plantar force distribution, Postoperative complication, Transfer metatarsalgia

## Abstract

**Background:**

Postoperative transfer metatarsalgia is a common complication after hallux valgus surgeries. Shortening of the first metatarsal is traditionally thought to be the primary cause of it. However, we speculate the abnormal loading pattern during gait is the real reason. This study is to determine specific differences in the loading patterns between reconstructive hallux valgus (HV) feet with and without postoperative transfer metatarsalgia, so as to find risky loading characteristics of this complication.

**Methods:**

Thirty feet with postoperative transfer metatarsalgia were recruited as pain group, while another 30 postoperative feet without pain as controls. All participants were asked to walk barefoot at self-selected speed through a plantar force measuring plate (Rs-Scan Inc.) for three times. Certain plantar load variables were recorded or calculated, and their differences between two groups were compared.

**Results:**

For pain group, the maximum plantar force and force time integral of the first metatarsal decrease significantly; the force time integral of the central rays (second plus third metatarsal) does not significantly differ with that in the controls, but their cumulative load percentage to the whole foot is higher. In pain group, the time point when central rays reached their peak force during the push-off is significantly later than that in controls. And the regional instant load percentage at this moment presented significantly higher for central rays, while significantly lower for the first metatarsal and the hallux compared to the controls.

**Conclusions:**

For hallux valgus feet with postoperative metatarsalgia, the load function of the first metatarsal is obviously impaired. But for central rays, indicative difference is not reflected in either peak or cumulative load during the gait cycle, but in the instant load distribution when central rays reach their peak load. So we can conclude that whether the remaining regions can adequately share certain load during walking, especially around the time metatarsalgia often occurs, plays an unnegligible role. So surgeons should pay more attention to reconstruct a foot where load can be evenly distributed.

## Background

Corrective surgery for hallux valgus (HV) deformities is one of the most common procedures in foot and ankle department. It is reported that more than 200,000 operations are performed per year to correct HV in the USA [1], but 25 to 33% cases may not be satisfied with their outcomes [2]. High unsatisfied rate is always related to postoperative complications, among which transfer metatarsalgia may be more prevalent than previously thought and sometimes unpredictable.

Shortening of the first ray has been thought to be the main cause of it. And according to Maestro et al. [3], the first metatarsal tended to be a similar length with the second or shorter than it within 2 mm in a harmony parabola arch, so surgeons always try to maintain an ideal metatarsal parabola during their procedures. However, enough soft tissue release and appropriate shortening are really needed in some circumstances, especially for severe deformities. Therefore, debate has emerged these years about whether and how much shortening of the first metatarsal could be allowed during HV correction, and different studies indicate different or even controversial conclusions [4–8].

However, the essential problem should not be the metatarsal length, and controversies on metatarsal length would still last if we continue focusing on the length itself, because the abnormal loading pattern secondary to inappropriate shortening might be the root cause indeed [9]. Other geometrical changes that may alter loading pattern during walking, such as elevation of the first metatarsal, supination of the forefoot, and subluxation of sesamoids, can also lead to transfer metatarsalgia. Therefore, the key point is about biomechanics, including dysfunction of the first ray and overload of lesser metatarsals. So a thorough understanding of the loading pattern in the foot with postoperative transfer metatarsalgia is a prerequisite, which would be followed by other studies on the relationship between different shortenings and the according loading pattern in the future, so as to solve the debate at last.

Galica et al. [10] tried to evaluate the plantar pressure and force of HV feet in a large population-based cohort, but they did not further classify the subjects according to different manifestation, which in fact might bring misinterpretation because different symptoms might couple with different or even contradictory loading patterns [11–14]. Wen et al. [13] divided the HV patients into pain and asymptomatic groups and investigated their difference in loading pattern with normal foot. But they did not distinguish between pain locations. Waldecker [15] compared HV feet with and without metatarsalgia and tried to find predictive pressure variabilities that are likely to result in metatarsalgia. However, preoperative HV feet, no matter with metatarsalgia or not, are biomechanically affected, but most of these biomechanical changes could be restored through correcting surgeries. So we do not think their findings about preoperative HV feet can predict postoperative transfer metatarsalgia.

Accordingly, the purpose of this study is to determine specific differences in the loading patterns between reconstructive HV feet with and without postoperative transfer metatarsalgia, so as to find risky loading characteristics of this complication and provide a biomechanical basis for future study about the real relationship between shortening and postoperative transfer metatarsalgia.

## Methods

### Sample size determination

Sample size considerations were based on available reports. According to previous studies [15] about the plantar pressure difference between HV feet with and without metatarsalgia, the mean difference on involved region was about 17 N/cm^2^ with a standard deviation of 20. If set the significant level at 0.05 and the statistical power at 0.9, a minimum of 30 cases was required for each group. The estimation procedures were performed by PASS (Power Analysis & Sample Size) software.

### Subjects

The study was approved by the institutional review board of our hospital. Patients who were surgically treated for their HV deformities at least 6 months ago were evaluated when they came back for routine check. Weight-bearing dorsoplantar and lateral radiographs of the fore foot were taken to make sure the postoperative alignment of each foot. The purpose, methods, and risks of the research were explained to all the potential participants. Nonunion or recurrent feet or those combined with pes cavus, rheumatoid arthritis, Morton’s neuroma, infection, or other unrelated foot pains were excluded.

According to the calculated sample size, 30 consecutive feet with postoperative transfer metatarsalgia during their daily walking were introduced and classified as group A. As controls, another consecutive 30 feet without metatarsalgia were recruited as group B. All participants gave their written informed consent. There is no significant difference between the two groups in age, body weight, gender, postoperative hallux valgus angle (HVA), or the first intermetatarsal angle (IMA) (Table 1). Transfer metatarsalgia in this study was defined as pain beneath the second to third metatarsals head during walking or intractable callosity with or even without pain, because callosity is an objective sign of transfer metatarsalgia except from subjective pain [16].

**Table 1:** General information of two groups of participants

	Group A	Group B
Age (year)	49.6 ± 14.6	51.4 ± 13.9
Body weight (kg)	55.6 ± 7.7	57.7 ± 8.5
Gender		
Male	4	7
Female	26	23
HVA (°)	9.1 ± 7.1	8.8 ± 6.6
1st IMA(°)	5.3 ± 2.6	4.9 ± 2.5

*HVA* hallux valgus angle, *1st IMA* first intermetatarsal angle

### Data collection

To collect plantar loading parameters, all participants were asked to walk barefoot at self-selected speed through a plantar force measuring plate (Rs-Scan Inc.) which was mounted flush in the middle of a 10-m-long carpet. The plate was 2 m × 0.4 m in dimension with a resolution of four sensors per square centimeter and sampling frequency of 250 Hz. For each foot, three valid trials were collected after some training and familiarization runs [17]. A valid trial of the tested foot should meet the following criteria:(1) presence of a complete footprint (2) with a heel-strike to toe-off gait pattern and (3) without visible adjustment when crossing the plate [18].

### Data analysis

In Rs-Scan software system, plantar area was usually automatically divided into 10 masks, including the hallux (T1), the second to fifth toes (T2–5), metatarsals 1–5 (M1 to M5, respectively), middle foot (MF), medial heel (HM), and lateral heel (HL) [19]. We then manually adjusted inappropriate divisions and, specifically, merged M2 and M3 into one mask (M2+3), as well as M4 and M5 into one (M4+5). These manual adjustments were confirmed following a standard procedure with a valid reliability [20].

For each tested foot and each divided region, plantar loading characteristics were calculated as follows: (1) peak force (PF) was defined as the maximum plantar force of each region during the gait cycle; (2) force time integral (FTI) was defined as the cumulative total force of each region over its contact time with the plate during the gait; (3) cumulative load percentage (CLP) was defined as the percentage of FTI of each region to the cumulative total force of the whole foot during the cycle; (4) instant load percentage (ILP) was defined as the percentage of the plantar force on each region to the total force of the foot at the time point that central metatarsals (i.e., M2+3) reached their PF during push-off phase; (5) peak time of central rays (PT_CR_) was defined as the percentage of the time that central metatarsals reached their PF to the whole standing phase period. Notably, the force mentioned above, no matter for each region or the whole foot, was the sum of all the sensors within the responding area.

Based on three valid trials, average values were calculated for each above parameters, whose differences between the two groups were then compared using independent two-tailed Student’s *t* tests. Alpha was set at 0.05, and differences with *P* values less than 0.05 were considered statistically significant. Statistical analysis was performed using SPSS 20.0 software (SPSS Inc., Chicago, USA).

## Results

**Fig. 1:**
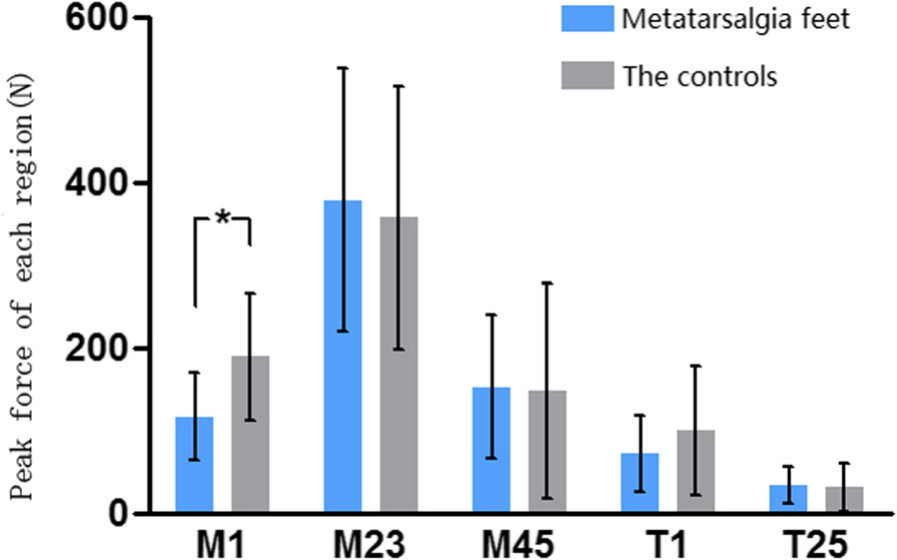
Differences of plantar peak force between the two groups on each forefoot region. Significant difference was only found on M1 region

For feet with postoperative transfer metatarsalgia (group A), mean peak force during the gait cycle was highest on M2+3 region, followed by M4+5, M1, T1, and T2–5, while for the controls (group B), the rank was almost the same except the reverse between M1 and M4+5. Significantly different PF between groups was observed only on M1 region with a lower PF in group A (*P* < 0.001), but cannot be found on other regions including M2+3 (*P* = 0.612) (Table 2, Fig. 1).

**Table 2:** Loading patterns of the forefoot between groups

		M1	M2+3	M4+5	T1	T2–5
PF (*N*)	Group A	116.5 ± 52.7	378.2 ± 158.8	152.9 ± 86.3	71.5 ± 45.8	33.8 ± 22.6
	Group B	189.5 ± 77.4	357.3 ± 158.3	148.1 ± 129.5	100.1 ± 77.3	31.6 ± 28.8
	*t*, *P*	−4.270, <0.001	0.511, 0.612	0.169, 0.866	−1.743, 0.087	0.329, 0.743
FTI (N S)	Group A	27.9 ± 17.1	114.1 ± 44.2	40.3 ± 27.3	17.7 ± 12.3	6.0 ± 3.3
	Group B	52.9 ± 23.1	101.5 ± 33.6	42.4 ± 22.3	25.9 ± 18.4	5.5 ± 3.9
	*t*, *P*	−4.764, <0.001	1.243, 0.219	−0.326, 0.745	−2.029, 0.047	0.536, 0.594
CLP (%)	Group A	8.4 ± 5.3	32.9 ± 9.1	11.4 ± 7.3	4.8 ± 5.8	1.8 ± 1.2
	Group B	14.1 ± 6.1	27.0 ± 6.8	11.1 ± 5.1	7.0 ± 5.3	1.6 ± 1.6
	*t*, *P*	−3.863, <0.001	2.845, 0.006	0.185, 0.854	−1.534, 0.131	0.548, 0.586
ILP (%)	Group A	13.4 ± 7.8	59.2 ± 9.7	18.8 ± 10.0	6.0 ± 5.6	2.6 ± 2.5
	Group B	24.5 ± 11.1	45.2 ± 9.3	13.5 ± 9.3	13.4 ± 7.6	3.4 ± 3.8
	*t*, *P*	−20.154, <0.001	5.706, <0.001	2.126, 0.038	−4.293, <0.001	−0.963, 0.339

*PF* peak force, *FTI* force time integral, *CLP* cumulative load percentage, *ILP* instant load percentage

**Fig. 2:**
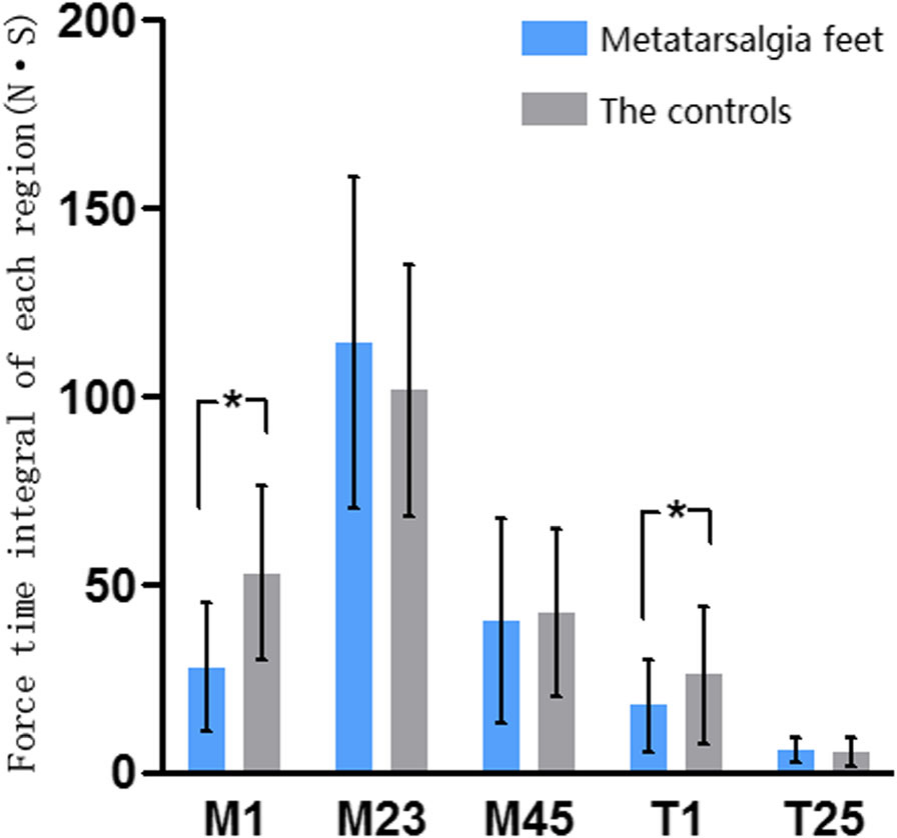
Differences of force time integral throughout gait cycle between the two groups on each forefoot region. Significant differences were found on both M1 and T1 regions

As for FTI, both M1 and T1 region of group A presented significantly lower values compared to group B, while M2+3 showed a higher tendency but without significance in group A. Significant difference was not found on other regions (Table 2, Fig. 2).

**Fig. 3:**
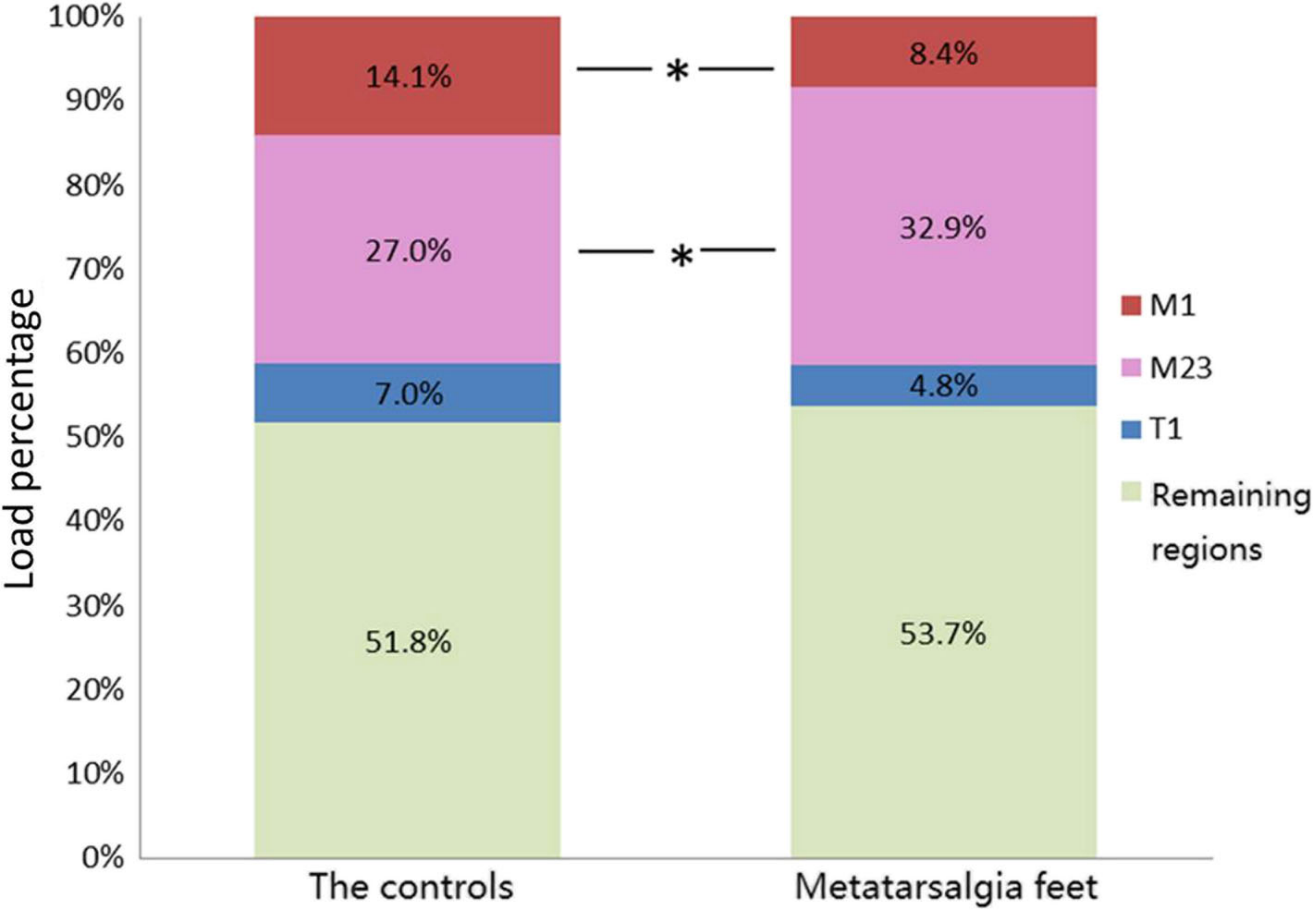
Differences of cumulative load percentage between the two groups on each forefoot region. Significant differences were found on both M1 and M2+3 regions

CLP was calculated based on FTI. Significant difference was only found on M1 and M2+3 between the two groups. Group A with metatarsalgia feet presented a lower CLP on M1 but higher one on M2+3, while the differences of other regions were not significant (Table 2, Fig. 3).

The duration of the standing phase was an average of 736.7 ± 114.0 ms in group A and 786.1 ± 136.0 ms in group B, without significant difference between them. The time point when central metatarsals (M2+3) reached their peak force was averagely at 623.5 ± 114.0 ms in group A, so the peak time of central rays (PT_CR_) was at 79.3% during the standing phase. In group B, however, the time point was at 569.1 ± 96.6 ms and the PT_CR_ was 77.2%. It demonstrated that central metatarsals in group A reached their peak force significantly later than those in group B (*P* = 0.037).

**Fig. 4:**
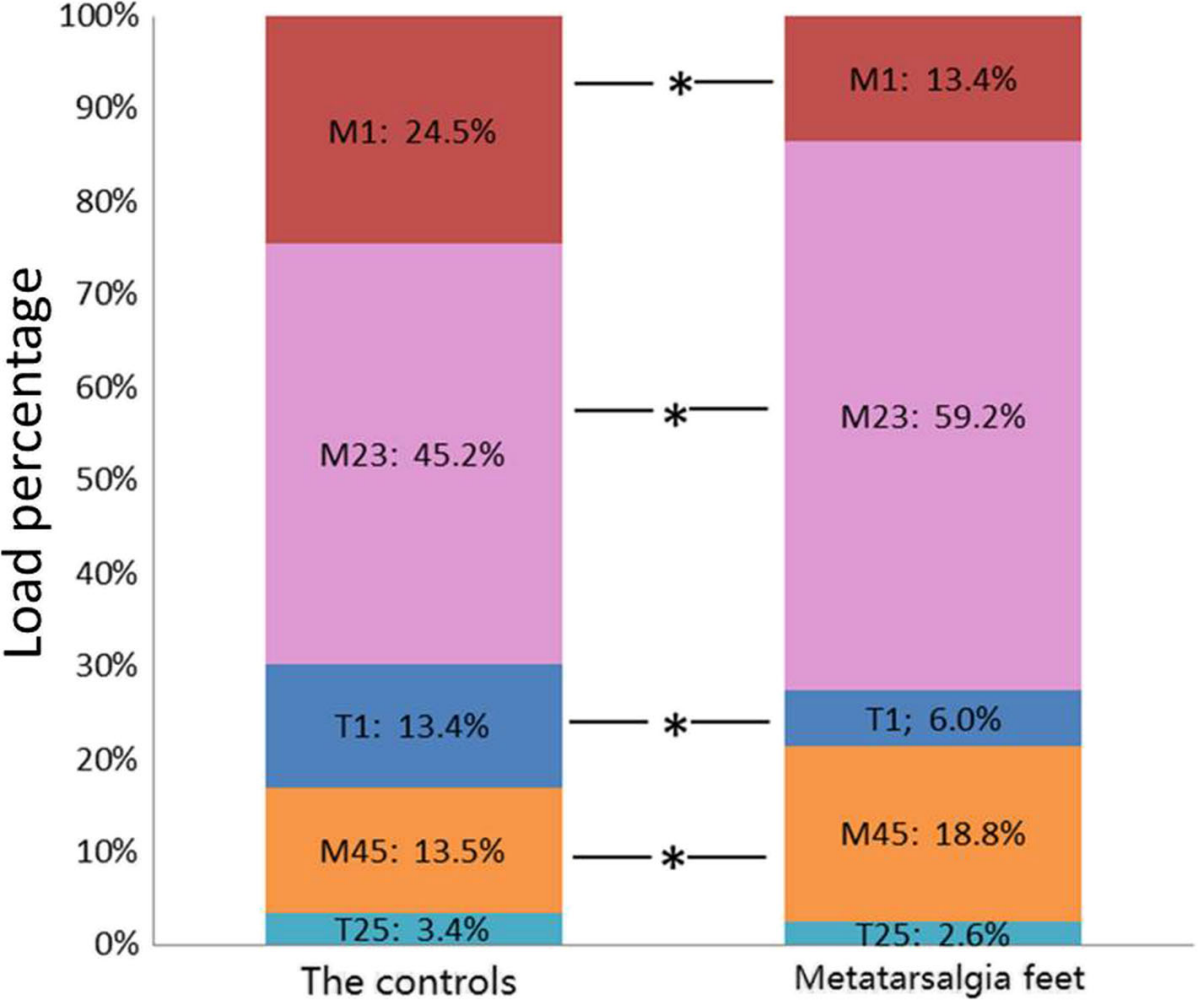
Differences of instant load percentage during the push-off between the two groups on each forefoot region. Significant differences were found on all regions except T2–5

At PT_CR_, furthermore, instant plantar force of each region was recorded, and each ILP was calculated. As a result, compared to group B, group A presented a significantly lower ILP on M1 or T1, while a higher one on M2+3. Difference in M4+5 between two groups was not that definite with the *P* value around 0.05 (Table 2, Fig. 4).

## Discussion

Postoperative transfer metatarsalgia is a common complication after HV reconstructive procedures, which often impairs patients’ final satisfaction. Shortening of the first metatarsal is traditionally thought to be the primary cause of iatrogenic metatarsalgia. However, we speculate the abnormal loading pattern during gait accompanied by shortening is the immediate cause, so we focus on it in this study.

Therefore, only postoperative HV patients were recruited in our study, and their plantar force distribution during gait cycle was compared between feet with and without transfer metatarsalgia, so as to investigate the specific loading distribution pattern for postoperative metatarsalgia. Considering that the second and third rays are the most common sites of metatarsalgia and it is sometimes difficult to differentiate between them, we chose to combine them into one region as central rays and the fourth and fifth metatarsals into one as lateral rays.

In the present study, significant difference in peak force between the two groups only lies in M1, which indicates that the maximum loading of the first ray is impaired for feet with postoperative metatarsalgia. Difference is not significant in central or lateral rays between two groups. However, maximum loading is only an instant variable which might emerge at different time point during gait cycle in different feet, which makes itself not necessarily a reliable parameter to compare between feet.

FTI reflects the whole loading volume during the gait cycle. The result shows M1 and T1 of metatarsalgia feet both bear significantly less weight throughout their gait, which further confirms the impaired function of the first ray and big toe. But other forefoot regions, even the pain-located central rays, all share similar load compared to non-metatarsalgia feet. We guess this may have relationship with relative less load of the whole forefoot.

This can be proved, on the one hand, by the peak time of central rays (PTCR), which shows central metatarsals in feet with metatarsalgia reach their peak force significantly later than those without pain. Combined with similar peak force and FTI, we can presume that the whole forefoot of metatarsalgia feet is likely to bear less weight and rely more on the hindfoot for loading during a gait cycle. This should be a gradually formed adaptive strategy to alleviate forefoot pain for those metatarsalgia feet. On the other hand, CLP can also confirm this point. Although absolute loading volumes of central rays were not significantly higher in metatarsalgia feet, their higher relative load percentages of the whole forefoot were found significant.

Galica et al. [10] reviewed up to 1123 HV feet and found they had lower maximum plantar force at big toe, first metatarsal, and lateral rays (second to fifth metatarsals) compared to normal feet. Although the sample size is very large, it included HV of all varieties and did not confine specific manifestation, which might lead to potential deviation. Lower maximum force at all metatarsals, in our opinion, implies ground contact of forefoot with HV might be delayed during a gait cycle.

Wen and his colleagues’ study [13] supported our finding that HV patients with pain tend to had shorter contact duration in most area of forefoot. Furthermore, they also found HV feet had significantly higher peak force in M2 and M3 compared to the normal and painful HV feet tend to load more in M1 and M2 compared to those without pain. These can be explained by that M1 and M2 are the most common pain-affected locations for HV feet before surgery. But our study suggests that, for postoperative HV feet with metatarsalgia, M2 and M3 tend to load relatively more while M1 and T1 have decreased loading. Therefore, all these indicate that local pain has direct relationship with increased loading.

Waldecker [15] reported that, compared to asymptomatic HV patients, both M1 and lateral rays (M2 to M5) in patients with metatarsalgia showed significantly greater peak pressure and pressure-time integral, while T1 tended to have lower values of these two parameters. Therefore, they believed that load transfer from T1, not M1, to lateral rays seemed to be a main cause of metatarsalgia in HV feet. However, from PF, FTI, and CLP in our study, decreased loading of M1 tends to play a more important role in the load transfer than T1. It is probably because the propulsive force of hallux is often restored when alignment is corrected after operation and the windlass mechanism is subsequently reconstructed [21]. Then, failure to restore appropriate loading of M1 contributes to relative load transfer to central rays and postoperative metatarsalgia.

Apart from the variables mentioned above, we additionally defined ILP which has not emerged in existing literature, which we deem may have more importance because pain occurs most often during pushing-off phase on central rays. We thus focus on the time point when central rays reach their peak loading and compare ILP of each region at this moment. Notably, patients with postoperative metatarsalgia showed definitely higher ILP in M2+3, while lower ILP in M1 and T1. It implies that whether M1 and T1 could share appropriate loading at the moment when central rays’ loading reaches its maximum plays an essential role in surgical outcome. More importantly, ILP is more comparable than PF due to confining to the same moment. And it seems to be more sensitive than other variables because difference of ILP between the two groups is significant on almost each region of forefoot.

Compared with previous similar studies, this study focuses on postoperative HV feet with single symptom of metatarsalgia, in which most abnormal biomechanics have been corrected unlike preoperative feet with various symptoms. Furthermore, instant load percentage at certain moment during the propulsive phase is found to be a sensitive prognostic factor, which has not been reported before.

Admittedly, several limits exist in this study. First, owing to relative small size, quantitative predictive variabilities are not calculated at last, although most significant differences are found between two groups; second, it is difficult for surgeons to avoid the loading pattern found in this study that may result in postoperative metatarsalgia, so future study are required about the relationship between metatarsal lengths and plantar force as well as how to control postoperative loading patterns during surgeries, thereby practically guiding HV procedures.

## Conclusions

For HV feet with postoperative metatarsalgia, the load function of the first metatarsal is obviously impaired. The cumulative load of central rays increased only relatively rather than absolutely during walking, because the forefoot contact duration of these feet is shorter. From the perspective of load balance of different plantar regions, instant load distribution at the moment when central rays reach their peak load is more important than regional maximum load itself. That is to say, whether the remaining regions can adequately share certain load plays unnegligible role. Therefore, as surgeons, we should pay more attention to reconstruct a foot where load can be evenly distributed especially when central rays reach their maximum during the push-off, rather than merely focusing on metatarsal lengths or idea parabola.

## Abbreviations

HV: Hallux valgus
HVA: Hallux valgus angle
IMA: Intermetatarsal angle
T1: Hallux
T2–5: The second to fifth toes
M1: The first metatarsal
M2+3: The second and third metatarsals
M4+5: The forth and fifth metatarsals
PF: Peak force
FTI: Force time integral
CLP: Cumulative load percentage
ILP: Instant load percentage
PT_CR_: Peak time of central rays.
